# Differences in medical costs among urban lung cancer patients with different health insurance schemes: a retrospective study

**DOI:** 10.1186/s12913-022-07957-9

**Published:** 2022-05-07

**Authors:** Yichen Li, Yong Yang, Jia Yuan, Lieyu Huang, Yong Ma, Xuefeng Shi

**Affiliations:** 1 West China Second University Hospital, Sichuan University, Chengdu, China; 2 Medical Device Regulatory Research and Evaluation Center, West China Hospital, Sichuan University, Chengdu, China; 3 School of Management, Beijing University of Chinese Medicine, No. 11, Bei San Huan Dong Lu, Chaoyang District, Beijing, 100029 People’s Republic of China; 4 West China Hospital, Sichuan University, Chengdu, China; 5 Office of Policy and Planning Research, Chinese Center for Disease Control and Prevention (China CDC), Beijing, China; 6 China Health Insurance Research Association, Beijing, China; 7 National Institute of Healthcare Security, Capital Medical University, Beijing, 100037 China; 8 National Institute of Traditional Chinese Medicine Strategy and Development, Beijing University of Chinese Medicine, Beijing, China

**Keywords:** Lung cancer, Health insurance, Health policy, Medical costs

## Abstract

**Background:**

Health insurance plays a significant role in reducing the financial burden for lung cancer patients. However, limited research exists regarding the differences in medical costs for lung cancer patients with different insurance schemes across different cities. We aimed to assess disparities in lung cancer patients’ costs by insurance type and city–specific insurance type.

**Methods:**

Claim data of China Urban Employees’ Basic Medical Insurance (UEBMI) and Urban Residents’ Basic Medical Insurance (URBMI) between 2010 and 2016 were employed to investigate differences in medical costs. This study primarily applied descriptive analysis and a generalized linear model with a gamma distribution and a log link.

**Results:**

In total, 92,856 lung cancer patients with inpatient records were identified, with Renminbi (RMB) 11,276 [6322–20,850] (median [interquartile range]) medical costs for the UEBMI group and RMB 8303 [4492–14,823] for the URBMI group. Out–of–pocket (OOP) expenses for the UEBMI group was RMB 2143 [1108–4506] and RMB 2975 [1367–6275] for the URBMI group. The UEBMI group also had significantly higher drug costs, medical service costs, and medical consumable costs, compared to the URBMI group. Regarding city-specific insurances, medical costs for the UEBMI and the URBMI lung cancer patients in Shanghai were RMB 9771 [5183–16,623] and RMB 9741 [5924–16,067], respectively. In Xianyang, the medical costs for UEBMI and URBMI patients were RMB 11,398 [6880–20,648] and RMB 9853 [5370–24,674], respectively. The regression results showed that the UEBMI group had 27.31% fewer OOP expenses than the URBMI group did, while patients in Xiangyang and Xianyang had 39.53 and 35.53% fewer OOP expenses, respectively, compared to patients in Shanghai.

**Conclusions:**

Compared with the URBMI patients, the UEBMI lung cancer patients obtained more or even better health services and had reduced financial burden. The differences in insurances among cities were greater, compared to those among insurances within cities, and the differences in OOP expenses between cities were greater compared to those between UEBMI and URBMI. Our results called for further reform of China’s fragmented insurance schemes.

**Supplementary Information:**

The online version contains supplementary material available at 10.1186/s12913-022-07957-9.

## Introduction

Lung cancer has been the second most commonly diagnosed cancer and the major cause of death from cancer worldwide, imposing a heavy disease burden on global health [1]. In 2018, the global number of new lung cancer cases was approximately 2.09 million, ranking first among all cancer types. Other than causing 19.4% of total cancer deaths, lung cancer is also considered one of the main causes of cancer–caused disability–adjusted life years (DALYs) [2, 3].

Lung cancer imposed a heavy burden on patients, their families, and the health system in China, with approximately 787,000 new cases in 2015. The age-standardized mortality rate in China reached 28.16 per 100,000 people, and approximately 30% of cancer deaths were due to lung cancer in 2015, both higher than most countries [4]. For each lung cancer patient in 2015, the average expenses in the first year following diagnosis accounted for 171% of the household annual income, and the all-direct expenses within 5 years after diagnosis was $42,540 [5]. For the whole country in the same year, about 0.6% of total health expenditure (RMB 24.31 billion) was on lung cancer treatment [6]. The incidence of catastrophic expenditure on lung cancer was estimated at 42.78%, higher than that for gastric, liver, esophageal, and breast cancer in China [7]. Under such circumstances, health insurance drew increasing public attention in that it could significantly get this burden down for families with lung cancer survivors.

China’s health insurance schemes for urban workers and urban residents bifurcate into the Urban Employee Basic Medical Insurance (UEBMI) and the Urban Resident Basic Medical Insurance (URBMI). UEBMI is compulsory and designed exclusively for urban employees. Contrarily, URBMI is a voluntary insurance program covering urban residents without formal employment, including young children, students, seniors, disabled, and other unemployed urban residents. The two health insurance schemes vary considerably in funding source, service coverage, and benefits packages [8]. Based on the annual salary of employees, employers and employees contribute 6 and 2% to UEBMI, respectively. URBMI is co-financed by both individuals and the government, the government has higher subsidies compared to individual premium contributions. In 2016, the per capital fund for UEBMI and URBMI was RMB 3478 and RMB 626, respectively [9]. UEBMI covers both outpatient and inpatient services whereas URBMI covers only inpatient services in most situations. Compared with URBMI, UEBMI provides a higher reimbursement rate, higher reimbursement ceiling, and more comprehensive service coverage, which means UEBMI has a better financial protection capacity for those enrolled. Importantly, a prior study stated that both UEBMI and URBMI schemes are pooled at the municipal level in China (approximately 333 UEBMI and 333 URBMI health insurance schemes under China’s fragmented health insurance system) [8], leading to uneven benefit packages in different insurance types and cities.

The financial protection ability of health insurance schemes is either an incentive or a disincentive for patients to utilize health services [10, 11]. Existing studies have compared patients’ medical costs for stroke [12], schizophrenia [13], and tuberculosis [14], supported by different insurance schemes. However, research investigating disparities in medical costs for lung cancer patients supported by different insurance schemes is scarce. Further, considering the differences in health insurance schemes among cities could be significant. Therefore, we used 7-year claims data for lung cancer from UEBMI and URBMI schemes in China to elucidate how these two health insurance schemes and their municipal differences shaped healthcare access and medical costs for lung cancer inpatients.

## Methods

### Data source

The data extracted claims from a 5% random sample comprising UEBMI and URBMI beneficiaries in 31 provinces in mainland China (covering more than 93% of the urban residents), who were supported by the China Health Insurance Research Association (CHIRA). A previous study describes the sampling process in detail [15]. The database includes 65 cities, all the records of urban beneficiaries’ demographic information, and primary diagnoses of hospital admissions. Thus, we believe that the data is accurate and reflecting the situations of all lung cancer patients in China. First, inpatient data for lung cancer between 2010 and 2016 from all included cities was used to analyze the overall differences in medical costs between these two health insurance groups. Second, data from three cities, Shanghai, Xiangyang, and Xianyang (referring to city A, B, and C, respectively), was used to compare medical costs by different economic levels (usually divided into eastern, central and western regions in China) through city-specific insurance schemes. All three cities had well-established health insurance systems, with a health insurance coverage that was higher than 95% before 2016. In 2019, the per capita GDP in China was RMB 72447, and the per capita GDP in Shanghai, Xiangyang, and Xianyang was RMB 157,300, RMB 84,700, and RMB 50,200, respectively. It is plausible that the differences in the lung cancer patients’ medical costs among all 333 cities could be inferred by assessing the differences in these three cities. According to the 10th revision of the International Statistical Classification of Disease (ICD-10), the principal diagnosis codes for all patients were identified as C34.

### Measures and variables

**Fig. 1:**
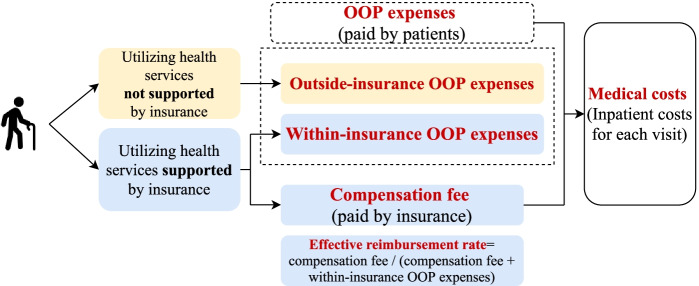
The main costs indicators in this study

Medical costs in the database comprised drug costs, medical service costs, and medical consumable costs. Other cost variables such as surgery and radiation therapy were also included in the medical costs, but not in detail. Drug costs could be further categorized as western medicine costs, Chinese patent medicine (CPM) costs, and Chinese herbal medicine (CHM) costs (The CPM and CHM belong to traditional Chinese medicine (TCM)). We used out-of-pocket (OOP) expenses per visit and outside–insurance OOP expenses per visit to reflect the financial burden of lung cancer patients. As the service package of UEBMI is more comprehensive, URBMI patients may face higher outside-insurance OOP expenses for the same health services, compared to the UEBMI patients. The effective reimbursement rate was among the important indicators of the financial protection ability and generosity of health insurance schemes. Figure 1 shows these four indicators and their relationships in detail. The control variables included gender, age group (younger than 45, 45–59, 60–75, and older than 75), hospital-level (primary, secondary, and tertiary), area (eastern, central, and western region), comorbidity (with or without comorbid conditions, which was identified using patients’ second diagnosis at discharge). Various types of comorbidities including cardiovascular disease, chronic obstructive pulmonary disease, diabetes mellitus, and hypertension were included), and year (from the year 2010 to2016). Control variables were only used when analyzing medical costs, OOP expenses, and outside-insurance OOP expenses. Our methods followed the guidelines for reporting economic evaluations (Consolidated Health Economic Evaluation Reporting Standards (CHEERS) statement) [16].

### Statistical analysis

This study mainly employed descriptive analysis and a generalized linear model (GLM) with a gamma distribution and a log link. First, median and interquartile range (IQR) were calculated to display medical costs, OOP expenses, outside-insurance OOP expenses by health insurance schemes, and control variables. Thereafter we evaluated the overall differences between the UEBMI and URBMI group regarding the four above-mentioned medical costs and the effective reimbursement rates. Second, we compared costs by health insurance schemes and cities. For the non-normal distribution of all cost variables, we used the Mann-Whitney test or Kruskal-Wallis test to inspect the differences in medical costs. Finally, GLM regression was employed to analyze the influence of the two insurance types and the cities with variance on medical costs. The STATA version 16.0 (Stata Corp, College Station, TX, USA) was mainly used to analyze the data and a *P*-value less than 0.05 was considered statistically significant.

## Results

### Sample characteristics

As shown in Table 1, this study involved 92,856 lung cancer patients with inpatient medical records (70,661 from UEBMI and 22,195 from URBMI) from 2010 to 2016. In both the UEBMI (47.88%) and the URBMI (49.07%) groups, the largest proportion was aged 60–75 years. Of the patients in the UEBMI groups, 75.43 and 50.61% chose tertiary hospitals for treatment, respectively. Nearly half the patients in both the UEBMI and the URBMI groups (49.84% vs. 46.03%) were from eastern China. The UEBMI group had more patients with comorbidity (27.04%) compared to the URBMI group (20.84%). By year, 22,767 patients (24.52%) were from 2016, 18,868 patients (20.32%) from 2015, 17,680 patients (19.04%) from 2014 and 17,022 (18.33%) patients from 2013. The patients between 2010 and 2012 totaled 16,519 (17.79%).

**Table 1:** Sample characteristics (*n* = 92,856)

	UEBMI	URBMI	Overall
**Gender**			
Male	48,496 (68.63)	13,004 (58.59)	61,500 (66.23)
Female	22,165 (31.37)	9191 (41.41)	31,356 (33.77)
**age**			
< 45	3104 (4.39)	1084 (4.88)	4188 (4.51)
45–59	20,977 (29.69)	6913 (31.15)	27,890 (30.04)
60–75	33,836 (47.88)	10,892 (49.07)	44,728 (48.17)
> 75	12,744 (18.04)	3306 (14.9)	16,050 (17.28)
**Hospital level**			
Primary	2668 (3.78)	2177 (9.81)	4845 (5.22)
Secondary	14,694 (20.8)	8785 (39.58)	23,479 (25.29)
Tertiary	53,299 (75.43)	11,233 (50.61)	64,532 (69.5)
**Region**			
East	35,220 (49.84)	10,216 (46.03)	45,436 (48.93)
Central	19,535 (27.65)	7113 (32.05)	26,648 (28.7)
West	15,906 (22.51)	4866 (21.92)	20,772 (22.37)
**Comorbidity**			
Yes	19,105 (27.04)	4625 (20.84)	23,730 (25.56)
No	51,556 (72.96)	17,570 (79.16)	69,126 (74.44)
**Year**			
2010	3675 (5.2)	590 (2.66)	4265 (4.59)
2011	5110 (7.23)	917 (4.13)	6027 (6.49)
2012	5247 (7.43)	980 (4.42)	6227 (6.71)
2013	13,725 (19.42)	3297 (14.85)	17,022 (18.33)
2014	13,480 (19.08)	4200 (18.92)	17,680 (19.04)
2015	13,319 (18.85)	5549 (25.00)	18,868 (20.32)
2016	16,105 (22.79)	6662 (30.02)	22,767 (24.52)
**Overall**	70,661 (76.10)	22,195 (23.9)	92,856

n (%) for all variables; UEBMI Urban Employees’ Basic Medical insurance; URBMI Urban Residents’ Basic Medical Insurance

### Medical costs for lung cancer patients by insurance type

Table 2 presents lung cancer patients’ medical costs, outside–insurance OOP expenses, and total OOP expenses, by different insurances. Overall, the UEBMI group had higher median medical costs than the URBMI group did (RMB 11,276 [6322 –20,850] vs. RMB 8303[4492–14,823]). Similar results were found (all *p* < 0.001) within the subgroups, especially hospital level and region (Supplementary Table 1). The overall median outside-insurance OOP expenses for the UEBMI group was lower than that for the URBMI group (RMB 302 [40–1430] vs. RMB 358 [60–1272]), and there were similar differences in most subgroups. However, regarding the mean value, the situation was reversed (RMB 2335 for the UEBMI and RMB 1782 for the URBMI, Supplementary Tables 2–3).

**Table 2:** Medical costs for lung cancer patients (RMB)

Indicators		UEBMI	URBMI	*P*–value
**Medical costs (RMB)**	**Hospital level**			
	Primary	5109 [2108–13,164]	2537 [1472–4693]	< 0.001
	Secondary	8697 [5035–15,709]	7148 [4217–12,641]	< 0.001
	Tertiary	12,433 [7115–22,732]	10,706 [6369–18,382]	< 0.001
	**Region**			
	East	11,345 [6209–20,620]	9433 [5237–16,299]	< 0.001
	Central	10,236 [5860–19,363]	7711 [4373–13,867]	< 0.001
	West	12,444 [72,44–23,230]	6834 [3250–13,025]	< 0.001
	**Overall**	11,276 [6322–20,850]	8303 [4492–14,823]	< 0.001
**Outside–insurance OOP expenses (RMB)**	**Hospital level**			
	Primary	45 [0.00–379]	51 [6–167]	0.275
	Secondary	160 [30–651]	320 [62–994]	< 0.001
	Tertiary	404 [53–1817]	565 [108–1927]	< 0.001
	**Region**			
	East	479 [30–2083]	833 [226–2152]	< 0.001
	Central	176 [40–763]	110 [24–441]	< 0.001
	West	300 [60–1083]	255 [57–764]	< 0.001
	**Overall**	302 [40–1430]	358 [60–1272]	< 0.001
**OOP expenses for lung cancer patients (RMB)**	**Hospital level**			
	Primary	713 [169–2017]	317 [117–779]	< 0.001
	Secondary	1512 [874–2846]	2292 [1219–4408]	< 0.001
	Tertiary	2451 [12,889–5214]	4389 [2365–8543]	< 0.001
	**Region**			
	East	2209 [1063–4914]	3987 [2102–7859]	< 0.001
	Central	2003 [1139–4049]	2774 [1577–5377]	< 0.001
	West	2189 [1143–4290]	1146 [332–3451]	< 0.001
	**Overall**	2143 [1108–4506]	2975 [1367–6275]	< 0.001
**Average number of hospitalization M (SD)**		1.92 (2.06)	1.88 (1.83)	< 0.001
**Median length of stay**		11 [6–17]	10 [6–15]	< 0.001

All costs results are displayed using Median [Interquartile Range]; M (SD) mean (standard deviation); UEBMI Urban Employees’ Basic Medical insurance; URBMI Urban Residents’ Basic Medical Insurance

Additionally, the URBMI group had significantly higher overall median OOP expenses than the UEBMI group did (RMB 2975 [1367–6275] vs. RMB 2143 [1108–4506]), and most subgroups showed similar results. However, in the subgroup including primary hospitals and the western region, the UEBMI patients had higher median OOP expenses, compared to the URBMI patients (Supplementary Table 4). Additionally, patients covered by UEBMI visited hospitals more frequently and stayed longer for treatment in hospitals, compared to those covered by URBMI.

### Differences in the composition of medical costs

**Fig. 2:**
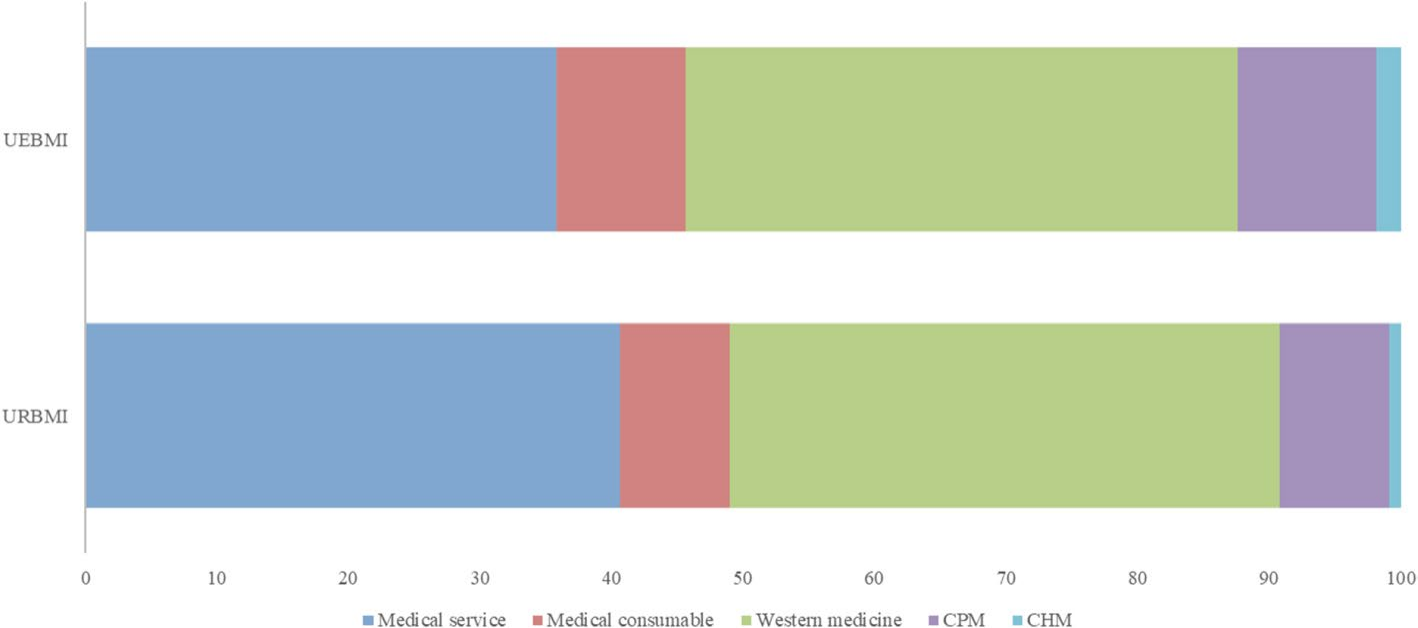
Compositions of medical costs for lung cancer patients with different insurances. CPM Chinese patent medicine; CHM Chinese herbal medicine; UEBMI Urban Employees’ Basic Medical insurance; URBMI Urban Residents’ Basic Medical Insurance

Table 3 and Fig. 2 present the differences between UEBMI and URBMI regarding the composition of medical costs for lung cancer patients. The UEBMI group had significantly higher median values of drug costs, TCM costs, medical service costs, and medical consumable costs than the URBMI group did (*p* < 0.001). For example, the UEBMI patients incurred higher median drug costs (RMB 6419 [3008–12,121]), compared to the URBMI patients (RMB 4477 [1943–8519]). Further, the UEBMI patients incurred higher median TCM costs than the URBMI patients did (RMB 945 [47–2497] vs. RMB 556 [22–1665]). Importantly, UEBMI had a higher effective reimbursement rate compared with URBMI.

**Table 3:** Composition of medical costs for lung cancer patients

	UEBMI	URBMI	Overall	*P*–value
Median drug cost (RMB)	6419	4477	5897	< 0.001
IQR	[3008–12,122]	[1943–8519]	[2679–11,270]	
Median TCM cost (RMB)	945	556	834	< 0.001
IQR	[47–2497]	[227–1665]	[36–2286]	
Median medical service cost (RMB)	3172	2603	3015	< 0.001
IQR	[1673–6821]	[1446–5286]	[1608–6407]	
Median medical consumable cost (RMB)	263	207	247	< 0.001
IQR	[90–765]	[70–560]	[84–709]	
Effective reimbursement rate	83.33%	65.61%	79.09%	< 0.001

*IQR* Interquartile range, *TCM* Traditional Chinese medicine (including Chinese patent medicine and Chinese herbal medicine), *UEBMI* Urban Employees’ Basic Medical insurance, *URBMI* Urban Residents’ Basic Medical Insurance

Regarding the proportion of different types of costs, the URBMI patients had higher medical service costs, but lower medical consumable and TCM costs, compared with the URBMI patients.

### Medical costs for lung cancer patients in three cities

Table 4 presents the differences in medical costs for lung cancer patients by city–specific health insurance schemes. City A had 4029 UEBMI and 375 URBMI patients, city B had 9829 UEBMI patients and 4334 URBMI patients, and city C had 640 UEBMI and 60 URBMI patients. The results revealed that the UEBMI patients had higher median medical costs and median outside–insurance OOP expenses, but lower median OOP expenses compared to the URBMI beneficiaries in all three cities. In Cities B and C, the UEBMI group had higher median drug costs compared with the URBMI group, and the median medical service costs for the UEBMI patients in cities A and B were higher than for the URBMI counterparts. Gaps of median medical costs between health insurance schemes among the cities were greater than the gaps within each city. For example, the median medical costs of either URBMI or URBMI in city A were higher than the corresponding median values in city B, but lower than those in city C. Similar gaps also existed in outside–insurance OOP cost, drug costs, medical service costs, medical consumable costs, and length of stay (LOS) indicators.

**Table 4:** Disparities in medical cost for patients in different cities

	City A		City B		City C		*P*–value
	UEBMI	URBMI	UEBMI	URBMI	UEBMI	URBMI	
N (%)	4029 (91.49)	375 (8.51)	9829 (69.4)	4334 (30.6)	640 (91.43)	60 (8.57)	
Median medical cost (RMB)	9–771	9–741	9091	7135	11,398	9853	< 0.001
IQR	[5183–16,623]	[5924–16,067]	[5570–16,864]	[4137–12,383]	[6880–20,648]	[5370–24,674]	
Median outside–insurance OOP cost (RMB)	210	166	70	54	186	130	< 0.001
IQR	[35–1040]	[48–774]	[15–246]	[6–192]	[52–499]	[52–326]	
Median OOP cost (RMB)	1909	3332	1626	2299	1636	2795	< 0.001
IQR	[934–3925]	[1905–6226]	[1034–2787]	[1426–4029]	[801–3635]	[1287–5758]	
Median drug cost (RMB)	5435	5771	5415	4027	6429	4233	< 0.001
IQR	[2750–10,402]	[3016–10,869]	[2879–9467]	[1789–7074]	[3553–10,581]	[1993–11,627]	
Median medical service cost (RMB)	3399	2684	2611	2309	3261	4621	< 0.001
IQR	[1290–6289]	[1474–5329]	[1530–5497]	[1445–4260]	[1845–8305]	[3024–8498]	
Median LOS	8	10	11	11	11	13	< 0.001
IQR	[3–11][4-11]	[5–16.25]	[4–15][7-18]	[4–14][7-17]	[7–19.25]	[8–20.5]	

IQR interquartile range; LOS length of stay; UEBMI Urban Employees’ Basic Medical insurance; URBMI Urban Residents’ Basic Medical Insurance

Table 5 showes the influence of health insurance schemes and cities on the expenditure of lung cancer patients. The UEBMI group had 26.24% (=exp^0.233^–1) higher medical costs than the URBMI group did, and patients in city C had 18.06% (=exp^0.166^–1) higher medical costs compared with patients in city A. For outside-insurance OOP expenses and OOP expenses, the differences between cities were greater than the differences between UEBMI and URBMI. Patients covered by UEBMI had 30.60% higher outside-insurance OOP expenses than patients covered by URBMI, while patients in city B had 84.15% (=1-exp^-1.842^) lower outside-insurance OOP expenses than patients in city A. The UEBMI group had 27.31% (=1-exp^− 0.319^) lower OOP expenses than the URBMI group, while a gap of 39.53% (=1-exp^-0.503^) for OOP expenses appeared between city A and city B and a gap of 35.53% (=1-exp^-0.439^) between city A and city C. After adding the urban per capita disposable income as a covariate, similar results were found (Supplementary Table 6).

**Table 5:** The impact of insurance type and city on patients’ cost

Characteristics	Medical costs		Outside-insurance OOP expenses		OOP expenses	
	Coef.	95% CI	Coef.	95% CI	Coef.	95% CI
Insurance type (Ref: URBMI)						
UEBMI	0.233^***^	[0.196,0.271]	0.267^***^	[0.134,0.400]	-0.319^***^	[−0.371,-0.267]
Cities (Ref:City A)						
City B	−0.044	[− 0.097,0.009]	−1.842^***^	[−2.052,-1.632]	− 0.503^***^	[− 0.576,-0.43]
City C	0.166^***^	[0.075,0.257]	−1.353^***^	[−1.678,-1.027]	−0.439^***^	[− 0.565,-0.314]

^***^ *p* < 0.01, ^**^ *p* < 0.05, ^*^ *p* < 0.1

*UEBMI* Urban Employees’ Basic Medical insurance, *URBMI* Urban Residents’ Basic Medical Insurance; All models were adjusted for gender, age group, hospital level, comorbidity, and year

## Discussion

Based on claims data from CHIRA, this study revealed differences in medical costs for lung cancer patients with two health insurance schemes, UEBMI and URBMI, and in different cities. Overall, compared with the URBMI group, the UEBMI group had higher medical costs (including drugs, medical service, and medical consumable) and higher mean outside-insurance OOP expenses, but lower OOP expenses, which means that the UEBMI group has utilized more comparatively (or more expensive) health services and bore less individual financial burden. The differences varied by cities—differences in insurances among cities were greater than the differences in insurances within cities. In addition, our study specifically showed that the differences in OOP expenses among cities were greater than the differences between UEBMI and URBMI. Regarding the overall outside–insurance OOP expenses, the mean and median values had opposite performance when comparing the UEBMI and URBMI. Using the median value, overall, UEBMI had lower outside–insurance OOP expenses than URBMI, but URBMI had higher ones when using the mean value. However, these two types of results were not contradictory. This is because compared to those in the URBMI groups, the upper-most quartiles of median outside-insurance OOP expenses in the UEBMI groups were higher; thus, despite more comprehensive service coverage of UEBMI a few patients with the UEBMI insurance scheme might have utilized health services that were out of range. Further, health services not supported by UEBMI were usually more expensive and of higher quality compared to those not covered in UEBMI.

The results regarding differences between UEBMI and URBMI were consistent with prior studies [17]. Using the China health and retirement longitudinal study (CHARLS) data, Wang et al. [18] reported that UEBMI had a greater effect in improving healthcare utilization and causing higher medical costs compared with URBMI. Based on claims data from Guangzhou province in China, Zhang et al. [19] found that the UEBMI dementia patients had higher hospitalization costs compared with the URBMI counterparts. Similarly, Chen et al. [20] revealed that the UEBMI diabetic patients incurred higher expenditure compared to the URBMI patients. The present finding that UEBMI lung cancer patients have lower OOP, was also similar to that of Yang et al. [12], who reported that the UEBMI stroke patients had fewer direct economic burdens than the URBMI counterparts. The differences in expenditure between the UEBMI and the URBMI patients were due to several possible reasons. From the patients’ socioeconomic status perspective, those in the UEBMI group were all urban workers or retired workers, compared with the disabled residents and unemployed patients who were covered by URBMI, usually having higher income (or pension), and better education. Income was an important contributor to healthcare utilization inequity, and people with high income had stronger incentive to utilize expensive health services and assume corresponding high medical costs [21, 22]. This was also the reason that some UEBMI patients had higher outside-insurance OOP expenses (paying medical services, drugs, and medical consumables which were not supported and compensated by the insurance schemes) compared with the URBMI patients in this study. As UEBMI’s service packages were more comprehensive compared to URBMI’s service packages, theabove-mentioned medical services, drugs, and medical consumables that were not supported by health insurance scheme were generally non-basic and expensive. The URBMI patients with low income could be more conservative when utilizing health services and drugs. Thus, differences in income between the UEBMI and the URBMI groups may lead to differences in medical costs. Further, we speculate that people with better education had more knowledge of health and were more willing to pay for it [23, 24], which attributed the UEBMI group’s higher medical costs to their better education.

From the perspective of the financial protection ability of insurance schemes, UEBMI provided a higher reimbursement rate and higher reimbursement ceiling compared to URBMI. Prior studies have proved that patients covered by health insurances with better financial protection tend to seek better quality health services in higher-level of hospitals [25]. The present results indicated that more patients in the UEBMI groups accepted treatment in tertiary hospitals, subsequently causing higher medical costs. Health insurance schemes with better financial protection had a greater effect on motivating patients to utilize more health services [19]. By disregarding catastrophic health expenditure, the UEBMI beneficiaries were more willing to use expensive drugs and medical consumables compared to the URBMI patients. In addition, patients with different insurances may choose different therapeutic schedules, naturally resulting in differences in the medical costs, drug costs, medical service costs, and medical consumable costs [26]. Conversely, doctors may also provide more reasonable treatments to reduce the economic burden for patients with URBMI [27]. The present findings regarding the differences in the composition of medical costs supported these two speculations indirectly. We believe that the higher reimbursement rate and higher reimbursed ceiling also caused the UEBMI patients to incur fewer OOP expenses than compared with URBMI patients. In brief, the UEBMI funding pool has incurred the highest medical costs for its beneficiaries, while URBMI funding pool was not as generous.

From the population characteristics perspective, the UEBMI group had more male patients than the URBMI group. Compared with female patients, male patients were more likely to smoke, which is the most threatening risk factor for lung cancer [28]. Therefore, male patients had a larger population attributable fraction (PAF) of lung cancer deaths caused by smoking, and higher medical costs compared to female patients [29, 30]. The higher medical costs for male lung cancer patients may have contributed to higher medical costs for the UEBMI group. Second, compared to the URBMI group, more patients in the UEBMI group had comorbidity, which has been proved to be significantly associated with high medical costs [31].

The presentstudy also foundthat the differences in medical costs among cities were greater than the differences in medical costs by insurances within cities; the same case applies to the OOP expenses. First, the different cities had separate UEBMI and URBMI funding pools, leading to the different service coverage and benefits packages. Hence the differences in financial protection between these two insurance schemes primarily played a role in the differences in medical costs and OOP expenses among cities [8]. Second, with different economic development levels, the UEBMI workers in different cities also had different levels of salaries, which caused the variance in medical costs and OOP expenses. Third, the prevalence of comorbidities such as hypertension and diabetes mellitus differed between cities. The comorbidity was associated with an increased risk of disease severity and medical costs [31–33]. Fourth, hospitals’health resources and medical technologies varied by cities in China, including the three cities above [34]. We believe that in some cities poor medical technology could have prolonged LOS for patients Table 4 showes that patients in city A had shorter median LOS than patients in city C, which proved this speculation directly. The prolonged LOS was significantly associated with medical costs [35].

In 2016, the Chinese government officially integrated URBMI and the new rural cooperative medical insurance (NCMS, initially designed for rural patients) to establish a unified health insurance scheme, Urban-Rural Residents Basic Medical Insurance (URRBMI), covering rural residents and those earlier covered by URBMI. Although URRBMI has significantly promoted equity in access to health care utilization especially for rural residents [36, 37], it did not significantly improve benefit packages for the original URBMI residents. Gaps between URBMI and UEBMI remain. Differences among lung cancer patients regarding medical costs and OOP expenses called for further integration of the fragmented insurance schemes in China. Notably, the current insurance integration in China was implemented within each municipal city, which improved the NCMS funding pools from county level to upper municipal level. A broader funding pool coudld resist economic risk more strongly [8]. The integration was conducive to changing the status quo of fragmented management involving health insurance schemes in China; however, it failed to counteract the role of income, or the presence of the UEBMI, in increasing inequality on healthcare utilization [38]. Our results indicate that the level of the UEBMI and URBMI funding pools could be further merged and improved to province level (even national level), providing residents with equal benefit packages and financial protection to reduce the gap between UEBMI and URBMI, and between different cities for lung cancer patients.

This study had several limitations. First, since URBMI and NCMS have been merged, a comparison between UEBMI and URRBMI could be a better choice. While the new insurance scheme did not drastically improve the benefit packages for the URBMI patients, the present results still reflect the differences between UEBMI and URBMI. Second, the claims data lacked clinical outcomes for lung cancer patients, thus, it was unclear whether UEBMI patients had a higher survival rate after paying higher medical costs. Third, this study did not include the indirect medical costs between the UEBMI and URBMI groups. Finally, owing to lacking detailed information regarding the cancer stage, histology type of lung cancer, and detailed lung cancer treatment, it was unclear how the cancer characteristics shaped the medical costs.

## Conclusion

This study offers a comprehensive evaluation of the differences in medical costs for lung cancer inpatients covered by different health insurance schemes. The UEBMI group was found to have higher medical costs, TCM costs, drug costs, medical consumable costs, and mean outside-insurance OOP expenses, but lower OOP expenses compared to the URBMI group. That is, the UEBMI patients have obtained more or better services and enjoyed less individual financial burden. In addition, differences in insurances among cities were greater than differences in insurances within cities, which were hitherto ignored. Under the health insurance schemes with different benefit packages, differences in OOP expenses between cities were higher compared with those between UEBMI and URBMI. The present results provide critical information for consolidating the fragmented insurance schemes in China and reducing differences between patients with different health insurance schemes.

## Supplementary Information

**Additional file 1.**

## Data Availability

The data are third-party data and were provided by China Health Insurance Research Association. Authors in this study have the right to use this dataset, but not the right to share and distribute. A de-identified minimal dataset of the quantitative data is available upon request to researchers who meet the criteria for confidential information, by sending a request to CHIRA.

## Abbreviations

UEBMI: Urban Employees’ Basic Medical Insurance
URBMI: Urban Residents’ Basic Medical Insurance
NCMS: New rural Cooperative Medical insurance
URRBMI: Urban-Rural Residents Basic Medical Insurance
TCM: Traditional Chinese medicine
CPM: Chinese patent medicine
CHM: Chinese herbal medicine
GLM: Generalized linear model
IQR: Interquartile range
OOP: Out-of-pocket

## References

[1] Sung H, Ferlay J, Siegel RL, Laversanne M, Soerjomataram I, Jemal A, et al. Global cancer statistics 2020: GLOBOCAN estimates of incidence and mortality worldwide for 36 cancers in 185 countries. CA Cancer J Clin. 2021;71(3):209-249. 10.3322/caac.21660 33538338

[2] Ferlay J, Soerjomataram I, Dikshit R, Eser S, Mathers C, Rebelo M (2015). Cancer incidence and mortality worldwide: sources, methods and major patterns in GLOBOCAN 2012. Int J Cancer.

[3] Soerjomataram I, Lortet-Tieulent J, Parkin DM, Ferlay J, Mathers C, Forman D (2012). Global burden of cancer in 2008: a systematic analysis of disability-adjusted life-years in 12 world regions. Lancet.

[4] Cao M, Li H, Sun D, Chen W (2020). Cancer burden of major cancers in China: a need for sustainable actions. Cancer Commun (Lond).

[5] Zhang X, Liu S, Liu Y, Du J, Fu W, Zhao X, et al. Economic burden for lung Cancer survivors in urban China. Int J Environ Res Public Health. 2017;14(3). 10.3390/ijerph14030308 PMC5369144 28294998

[6] Yue C, Baohu Y, Gongwei Z (2018). Analysis of direct economic burden and average hospitalization cost of lung cancer in China in 2011–2015. Chin J Health Stat.

[7] Zheng A, Duan W, Zhang L, Bao X, Mao X, Luo Z (2018). How great is current curative expenditure and catastrophic health expenditure among patients with cancer in China? A research based on “system of health account 2011”. Cancer Med.

[8] Meng Q, Fang H, Liu X, Yuan B, Xu J (2015). Consolidating the social health insurance schemes in China: towards an equitable and efficient health system. Lancet.

[9] China MoHRaSSotPsRo (2017). China labour statistical yearbook 2017.

[10] Aryeetey GC, Westeneng J, Spaan E, Jehu-Appiah C, Agyepong IA, Baltussen R (2016). Can health insurance protect against out-of-pocket and catastrophic expenditures and also support poverty reduction? Evidence from Ghana's National Health Insurance Scheme. Int J Equity Health.

[11] Ta Y, Zhu Y, Fu H (2020). Trends in access to health services, financial protection and satisfaction between 2010 and 2016: has China achieved the goals of its health system reform?. Soc Sci Med.

[12] Yang Y, Man X, Nicholas S, Li S, Bai Q, Huang L (2020). Utilisation of health services among urban patients who had an ischaemic stroke with different health insurance - a cross-sectional study in China. BMJ Open.

[13] Zhang H, Sun Y, Zhang D, Zhang C, Chen G (2018). Direct medical costs for patients with schizophrenia: a 4-year cohort study from health insurance claims data in Guangzhou city, southern China. Int J Ment Heal Syst.

[14] Mao W, Jiang W, Hamilton C, Zhang H, Huang F, Lucas H (2019). Over- and under-treatment of TB patients in eastern China: an analysis based on health insurance claims data. Tropical Med Int Health.

[15] Yong M (2018). Research on medical expenses and influencing factors of stroke patients in Chinese urban residents [博士].

[16] Husereau D, Drummond M, Petrou S, Carswell C, Moher D, Greenberg D (2013). Consolidated health economic evaluation reporting standards (CHEERS) statement. BMC Med.

[17] Bai L, Wushouer H, Huang C, Luo Z, Guan X, Shi L (2020). Health care utilization and costs of patients with prostate cancer in China based on National Health Insurance Database from 2015 to 2017. Front Pharmacol.

[18] Wang Z, Li X, Chen M, Si L (2018). Social health insurance, healthcare utilization, and costs in middle-aged and elderly community-dwelling adults in China. Int J Equity Health.

[19] Zhang H, Zhang D, Yin Y, Zhang C, Huang Y. Costs of hospitalization for dementia in urban China: estimates from two urban health insurance scheme claims data in Guangzhou City. Int J Environ Res Public Health. 2019;16(15). 10.3390/ijerph16152781 PMC6695624 31382609

[20] Chen C, Song J, Xu X, Zhou L, Wang Y, Chen H (2020). Analysis of influencing factors of economic burden and medical service utilization of diabetic patients in China. PLoS One.

[21] Barraza-Lloréns M, Panopoulou G, Díaz BY (2013). Income-related inequalities and inequities in health and health care utilization in Mexico, 2000-2006. Rev Panam Salud Publica.

[22] Vásquez F, Paraje G, Estay M (2013). Income-related inequality in health and health care utilization in Chile, 2000–2009. Rev Panam Salud Publica.

[23] Schulz M (2017). The intertwined relationship between patient education, hospital waiting times and hospital utilization. Health Serv Manag Res.

[24] Gróf M, Vagašová T, Oltman M, Skladaný Ľ, Maličká L (2017). Inequalities in Cancer deaths by age, gender and education. Cent Eur J Public Health.

[25] Yang Y, Li S, Wang X, Guo Y, Ma Y, Shi X (2020). The utilization of inpatient health services and influencing factors of hospitalization costs of urban stroke patients in China. Chin Gen Pract.

[26] Johansen KL, Zhang R, Huang Y, Patzer RE, Kutner NG (2012). Association of race and insurance type with delayed assessment for kidney transplantation among patients initiating dialysis in the United States. Clin J Am Soc Nephrol.

[27] Medford-Davis LN, Fonarow GC, Bhatt DL, Xu H, Smith EE, Suter R (2016). Impact of insurance status on outcomes and use of rehabilitation Services in Acute Ischemic Stroke: findings from get with the guidelines-stroke. J Am Heart Assoc.

[28] Tuvdendorj A, Feenstra T, Tseveen B, Buskens E (2020). Smoking-attributable burden of lung cancer in Mongolia a data synthesis study on differences between men and women. Plos One.

[29] Zheng W, McLerran DF, Rolland BA, Fu Z, Boffetta P, He J (2014). Burden of total and cause-specific mortality related to tobacco smoking among adults aged ≥ 45 years in Asia: a pooled analysis of 21 cohorts. Plos Med.

[30] Akbari Sari A, Rezaei S, Arab M, Karami Matin B, Majdzadeh R (2017). Does smoking status affect cost of hospitalization? Evidence from three main diseases associated with smoking in Iran. Med J Islam Repub Iran.

[31] Ding R, Zhu D, He P, Ma Y, Chen Z, Shi X (2020). Comorbidity in lung cancer patients and its association with medical service cost and treatment choice in China. BMC Cancer.

[32] Wang Z, Chen Z, Zhang L, Wang X, Hao G, Zhang Z (2018). Status of hypertension in China: results from the China hypertension survey, 2012-2015. Circulation.

[33] Sun J, Ji J, Wang Y, Gu HF (2021). Association of the Haze and Diabetes in China. Curr Diabetes Rev.

[34] Fan C, Ouyang W, Tian L, Song Y, Miao W. Elderly health inequality in China and its determinants: a geographical perspective. Int J Environ Res Public Health. 2019;16(16). 10.3390/ijerph16162953 PMC6719074 31426371

[35] Yu T, He Z, Zhou Q, Ma J, Wei L (2015). Analysis of the factors influencing lung cancer hospitalization expenses using data mining. Thorac Cancer.

[36] Liu H, Zhu H, Wang J, Qi X, Zhao M, Shan L (2019). Catastrophic health expenditure incidence and its equity in China: a study on the initial implementation of the medical insurance integration system. BMC Public Health.

[37] Li C, Tang C, Wang H (2019). Effects of health insurance integration on health care utilization and its equity among the mid-aged and elderly: evidence from China. Int J Equity Health.

[38] Zhao M, Liu B, Shan L, Li C, Wu Q, Hao Y (2019). Can integration reduce inequity in healthcare utilization? Evidence and hurdles in China. BMC Health Serv Res.

