# Correction: Differences in medical costs among urban lung cancer patients with different health insurance schemes: a retrospective study

**DOI:** 10.1186/s12913-022-08083-2

**Published:** 2022-05-19

**Authors:** Yichen Li, Yong Yang, Jia Yuan, Lieyu Huang, Yong Ma, Xuefeng Shi

**Affiliations:** 1 West China Second University Hospital, Sichuan University, Chengdu, China; 2 Medical Device Regulatory Research and Evaluation Center, West China Hospital, Sichuan University, Chengdu, China; 3 School of Management, Beijing University of Chinese Medicine, No. 11, Bei San Huan Dong Lu, Chaoyang District, Beijing, 100029 People’s Republic of China; 4 West China Hospital, Sichuan University, Chengdu, China; 5 Office of Policy and Planning Research, Chinese Center for Disease Control and Prevention (China CDC), Beijing, China; 6 China Health Insurance Research Association, Beijing, China; 7 National Institute of Healthcare Security, Capital Medical University, Beijing, 100037 China; 8 National Institute of Traditional Chinese Medicine Strategy and Development, Beijing University of Chinese Medicine, Beijing, China

**Correction: BMC Health Serv Res 22, 612 (2022)**

**https://doi.org/10.1186/s12913-022-07957-9**

Following publication of the original article [1], the authors reported missing information in the ‘Funding’ section.

The statement in the ‘Funding’ section originally read: No funding was obtained for this study.

The statement in the ‘Funding’ section should read: This study was supported by the China National Health Development Research Center (grant number 2020QT-CNHDRC-2020-28).

The original article [1] has been updated.
