# Supplementary material for: Differences in medical costs among urban lung cancer patients with different health insurance schemes: a retrospective study
Source: BMC Health Serv Res. 2022 May 7;22:612. doi: 10.1186/s12913-022-07957-9 (PMC9077891; doi:10.1186/s12913-022-07957-9)
Supplement: Supplementary file 1 — Additional file 1. [file 12913_2022_7957_MOESM1_ESM.docx]

**Supplementary Tables**

**Table 1 Medical costs for lung cancer patients (RMB)**

|  | UEBMI | URBMI | P-value | | |
| --- | --- | --- | --- | --- | --- |
| **Gender** |  |  |  | | |
| Male | 11398.87[6469.39,20946.20] | 8338.18[4419.16,14935.5] | <0.001 | | |
| Female | 10954.47[6008.75,20550.42] | 8226.70[4600.90,14706.37] | <0.001 | | |
| **age** |  |  |  | | |
| <45 | 12007.09[6444.31,23560.43] | 9143.83[4862.77,16538.29] | <0.001 | | |
| 45-59 | 11275.91[6346.68,20770.84] | 8733.83[4863.14,15461.08] | <0.001 | | |
| 60-74 | 11219.84[6376.32,20590.21] | 8199.44[4469.67,14726.85] | <0.001 | | |
| >75 | 11205.03[6121.40,21134.50] | 7338.49[3783.00,13456.87] | <0.001 | | |
| **Comorbidity** |  |  |  | | |
| Yes | 13106.79[7005.3,23738.19] | 10781.80[5936.83,18643.74] | <0.001 | | |
| No | 10701.12[6123.99,19838.60] | 7704.34[4202.06,13876.91] | <0.001 | | |
| **Year** |  |  |  | | |
| 2010 | 10716.74[6081.01,19508.17] | 8718.11[4965.71,15008.1] | <0.001 | | |
| 2011 | 10556.62[5948.74,18380.8] | 9431.44[4940.80,15789.51] | <0.001 | |  |
| 2012 | 12208.92[6475.73,20838.36] | 8755.23[4806.40,15733.53] | <0.001 | |  |
| 2013 | 13469.76[7353.56,25578.95] | 9446.96[5001.23,16975.9] | <0.001 | |  |
| 2014 | 10895.13[6721.39,19967.33] | 8129.76[4327.63,14481.56] | <0.001 | |  |
| 2015 | 11005.03[6142.32,20602.82] | 7638.20[4151.25,13874.20] | <0.001 | |  |
| 2016 | 10237.81[5472.02,19336.61] | 8210.20[4522.38,14734.12] | <0.001 | |  |
| **Overall** | 11276.44[6322.06,20850.19] | 8302.95[4491.95,14822.60] | <0.001 |  |  |

All results are displayed using Median [Interquartile Range], UEBMI Urban Employees’ Basic Medical insurance; URBMI Urban Residents’ Basic Medical Insurance

**Table** **2 Outside-insurance OOP expenses (RMB)**

|  | UEBMI | URBMI | P-value |
| --- | --- | --- | --- |
| **Gender** |  |  |  |
| Male | 306.45[43.00,1395.25] | 369.39[63.69,1266.04] | <0.001 |
| Female | 294.43[33.00,1521.94] | 349.29[56.03,1279.23] | 0.001 |
| **age** |  |  |  |
| <44 | 420.18[55.00,2202.81] | 309.63[63.14,1355.01] | 0.011 |
| 45-59 | 309.30[39.60,1482.00] | 395.88[68.97,1364.85] | <0.001 |
| 60-74 | 296.65[40.00,1425.66] | 356.53[60.18,1267.37] | <0.001 |
| >75 | 292.25[41.48,1269.63] | 300.03[44.54,1074.00] | 0.316 |
| **Comorbidity** |  |  |  |
| Yes | 741.85[94.00,2858.75] | 677.88[98.50,2306.48] | <0.001 |
| No | 221.04[31.00,1050.32] | 308.52[55.00,1074.17] | <0.001 |
| **Year** |  |  |  |
| 2010 | 390.09[87.40,1467.53] | 492.95[149.76,1476.53] | 0.008 |
| 2011 | 363.53[88.44,1101.29] | 375.00[105.11,1352.70] | 0.160 |
| 2012 | 470.00[81.33,1549.63] | 423.64[74.01,1371.12] | 0.274 |
| 2013 | 556.00[109.00,2097.90] | 625.00[149.65,1772.20] | 0.117 |
| 2014 | 130.78[0.00,939.51] | 315.57[44.00,1108.21] | <0.001 |
| 2015 | 237.62[28.70,1266.99] | 236.87[39.00,1045.52] | 0.528 |
| 2016 | 275.39[25.00,1498.22] | 353.56[63.00,1292.62] | <0.001 |
| **Overall** | 302.05[40.00,1430] | 358.00[60.18,1272.40] | <0.001 |

All results are displayed using Median [Interquartile Range], UEBMI Urban Employees’ Basic Medical insurance; URBMI Urban Residents’ Basic Medical Insurance

**Table 3 Outside-insurance OOP expenses for lung cancer patients (RMB)**

|  | UEBMI | URBMI | P-value |
| --- | --- | --- | --- |
| **Gender** |  |  | 0.258 |
| Male | 2243.21±9196.02 | 1842.74±8413.46 | <0.001 |
| Female | 2537.25±8165.49 | 1697.16±4835.51 | 0.001 |
| **age** |  |  | <0.001 |
| <45 | 3694.99±20203.97 | 1885.88±4947.52 | 0.011 |
| 45-59 | 2519.51±8672.34 | 2106.48±9472.65 | <0.001 |
| 60-74 | 2298.83±8329.14 | 1752.05±6484.42 | <0.001 |
| >75 | 1798.55±5449.83 | 1171.17±2950.65 | 0.316 |
| **Hospital level** | |  | <0.001 |
| Primary | 1206.45±7963.23 | 358.67±2983.24 | 0.275 |
| Secondary | 1043.79±3998.82 | 1073.87±2821.08 | <0.001 |
| Tertiary | 2748.06±9819.48 | 2612.55±9574.24 | <0.001 |
| **Region** |  |  | <0.001 |
| East | 2848.14±6946.34 | 2291.97±4552.32 | <0.001 |
| Central | 1811.12±13133.33 | 1322.71±10589.3 | <0.001 |
| West | 1844.18±5567.57 | 1384.79±4993.76 | <0.001 |
| **Comorbidity** |  |  | <0.001 |
| Yes | 3636.76±14130.82 | 3166.27±13414.86 | <0.001 |
| No | 1853.22±5777.93 | 1418.19±4077.58 | <0.001 |
| **Year** |  |  | <0.001 |
| 2010 | 1772.93±4507.59 | 1601.63±3661.83 | 0.008 |
| 2011 | 1646.25±4744.55 | 1637.85±4050.42 | 0.160 |
| 2012 | 2208.71±5656.67 | 1951.65±4752.87 | 0.274 |
| 2013 | 2722.88±6865.09 | 2155.15±4526.29 | 0.117 |
| 2014 | 1955.93±5709.84 | 1498.07±4077.63 | <0.001 |
| 2015 | 2716.45±15964.74 | 1863.83±11981 | 0.528 |
| 2016 | 2396.17±6743.56 | 1720.55±4835.3 | <0.001 |
| **Overall** | 2335.45±8886.63 | 1782.46±7152.57 | <0.001 |

All values are displayed using mean±standard deviation; UEBMI Urban Employees’ Basic Medical insurance; URBMI Urban Residents’ Basic Medical Insurance;

**Table 4 OOP expenses for lung cancer patients (RMB)**

|  | UEBMI | URBMI | P-value |
| --- | --- | --- | --- |
| **Gender** |  |  |  |
| Male | 2161.81[1125.03,4447.65] | 2879.47[1247.99,6204.19] | <0.001 |
| Female | 2103.17[1066.81,4659.93] | 3094.81[1521.12,6339.92] | <0.001 |
| **age** |  |  |  |
| <44 | 2729.92[1343.47,6590.14] | 3336.75[1625.63,7043.94] | 0.001 |
| 45-59 | 2278.84[1166.17,4921.08] | 3195.08[1520,6799.83] | <0.001 |
| 60-74 | 2082.59[1085.68,4301.81] | 2919.17[1347.45,6167.91] | <0.001 |
| >75 | 1983.59[1023.17,4009.1] | 2496.07[1004.93,5321.58] | <0.001 |
| **Comorbidity** |  |  |  |
| Yes | 2796.32[1341.94,6223.46] | 4900[2443.92,9229.33] | 0.004 |
| No | 1966.8[1043.79,3989.61] | 2609.66[1205.05,5369.97] | <0.001 |
| **Year** |  |  |  |
| 2010 | 2374.36[1291.9,4458.67] | 4084.5[2379.88,7616.8] | <0.001 |
| 2011 | 2071.82[1127.4,3827.44] | 3546.21[1762.66,6712.09] | <0.001 |
| 2012 | 2444.48[1250.37,4712.16] | 3668.51[1765.26,7021.35] | <0.001 |
| 2013 | 2641.88[1349.14,5877.17] | 3628.95[1603.44,7913.97] | <0.001 |
| 2014 | 1920.78[1116.43,3784.74] | 2524.41[1250.4,5476.19] | <0.001 |
| 2015 | 2115.38[1146.87,4460.83] | 2590.73[1097.98,5707.15] | <0.001 |
| 2016 | 1862.7[782,4255.96] | 2992.7[1445.97,6123.2] | <0.001 |
| **Overall** | 2143.08[1107.90,4505.96] | 2975.14[1367.03,6275.09] | <0.001 |

All results are displayed using Median [Interquartile Range], UEBMI Urban Employees’ Basic Medical insurance; URBMI Urban Residents’ Basic Medical Insurance

**Table 5 The impact of insurance type and city on patients’ cost**

| Characteristics | | Medical costs | | Outside-insurance OOP expenses | | OOP expenses | |
| --- | --- | --- | --- | --- | --- | --- | --- |
|  |  | Coef. | 95% CI | Coef. | 95% CI | Coef. | 95% CI |
| Insurance type (Ref: URBMI) | | | |  |  |  |  |
| UEBMI | | 0.233^***^ | [0.196,0.271] | 0.267^***^ | [0.134,0.400] | -0.319^***^ | [-0.371,-0.267] |
| Cities (Ref:City A) | | |  |  |  |  |  |
| City B | | -0.044 | [-0.097,0.009] | -1.842^***^ | [-2.052,-1.632] | -0.503^***^ | [-0.576,-0.43] |
| City C | | 0.166^***^ | [0.075,0.257] | -1.353^***^ | [-1.678,-1.027] | -0.439^***^ | [-0.565,-0.314] |
| Gender (Ref: Female) | | |  |  |  |  |  |
| Male | 0.049^***^ | | [0.016,0.082] | 0.046 | [-0.072,0.164] | 0.028 | [-0.017,0.074] |
| Age group (Ref: <44) | | |  |  |  |  |  |
| 45-59 | -0.110^**^ | | [-0.195,-0.026] | -0.183 | [-0.484,0.118] | -0.126^**^ | [-0.243,-0.01] |
| 60-74 | -0.134^***^ | | [-0.217,-0.052] | -0.272^*^ | [-0.566,0.022] | -0.163^***^ | [-0.277,-0.05] |
| >75 | -0.124^***^ | | [-0.213,-0.034] | -0.399^**^ | [-0.719,-0.08] | -0.211^***^ | [-0.335,-0.088] |
| Hospital level (Ref: Primary) | | | |  |  |  |  |
| Secondary | 0.688^***^ | | [0.6,0.776] | 1.185^***^ | [0.849,1.521] | 0.645^***^ | [0.522,0.768] |
| Tertiary | 0.859^***^ | | [0.774,0.945] | 1.619^***^ | [1.309,1.93] | 0.950^***^ | [0.832,1.068] |
| Comorbidity (Ref: No) | | |  |  |  |  |  |
| Yes | 0 | | - | 0 | - | 0 | - |
| Year (Ref: 2010) | | |  |  |  |  |  |
| 2011 | -0.048 | | [-0.163,0.066] | -0.183 | [-0.589,0.224] | 0.033 | [-0.126,0.191] |
| 2012 | -0.168^***^ | | [-0.281,-0.054] | 0.036 | [-0.374,0.445] | 0.058 | [-0.1,0.216] |
| 2013 | -0.011 | | [-0.140,0.118] | -0.047 | [-0.506,0.412] | 0.026 | [-0.151,0.204] |
| 2014 | -0.026 | | [-0.133,0.081] | 0.064 | [-0.321,0.449] | 0.121 | [-0.026,0.269] |
| 2015 | 0.046 | | [-0.061,0.152] | 0.23 | [-0.157,0.617] | 0.246^***^ | [0.099,0.393] |
| 2016 | 0.094^*^ | | [-0.012,0.199] | 0.683^***^ | [0.291,1.075] | 0.202^***^ | [0.057,0.348] |

^***^ p<0.01, ^**^ p<0.05, ^*^ p<0.1; UEBMI Urban Employees’ Basic Medical insurance; URBMI Urban Residents’ Basic Medical Insurance; All models were adjusted for gender, age group, hospital level, comorbidity, and year.

**Table 6 The impact of insurance type and city on patients’ cost (adding** **urban per capita disposable income as a covariate)**

| Characteristics | | Medical costs | | Outside-insurance OOP expenses | | OOP expenses | |
| --- | --- | --- | --- | --- | --- | --- | --- |
|  |  | Coef. | 95% CI | Coef. | 95% CI | Coef. | 95% CI |
| Insurance type (Ref: URBMI) | | | |  |  |  |  |
| UEBMI | | 0.231^***^ | [0.194,0.269] | 0.268^***^ | [0.135,0.402] | -0.321^***^ | [-0.373,-0.269] |
| Cities (Ref:City A) | | |  |  |  |  |  |
| City B | | 0.324^*^ | [-0.033,0.681] | -2.589^***^ | [-3.987,-1.191] | -0.100 | [-0.603,0.402] |
| City C | | 0.421^***^ | [0.159,0.683] | -1.851^***^ | [-2.822,-0.88] | -0.165 | [-0.528,0.199] |
| Gender (Ref: Female) | | |  |  |  |  |  |
| Male | 0.049^***^ | | [0.016,0.082] | 0.046 | [-0.071,0.164] | 0.028 | [-0.017,0.074] |
| Age group (Ref: <44) | | |  |  |  |  |  |
| 45-59 | -0.109^**^ | | [-0.194,-0.025] | -0.185 | [-0.487,0.116] | -0.125^**^ | [-0.241,-0.009] |
| 60-74 | -0.134^***^ | | [-0.217,-0.052] | -0.272^*^ | [-0.567,0.022] | -0.163^***^ | [-0.277,-0.049] |
| >75 | -0.122^***^ | | [-0.212,-0.033] | -0.409^**^ | [-0.729,-0.089] | -0.208^***^ | [-0.331,-0.085] |
| Hospital level (Ref: Primary) | | | |  |  |  |  |
| Secondary | 0.698^***^ | | [0.609,0.787] | 1.173^***^ | [0.837,1.509] | 0.66^***^ | [0.535,0.784] |
| Tertiary | 0.87^***^ | | [0.784,0.956] | 1.607^***^ | [1.297,1.918] | 0.965^***^ | [0.846,1.084] |
| Comorbidity (Ref: No) | | |  |  |  |  |  |
| Yes | 0 | | - |  |  |  |  |
| Year (Ref: 2010) | | |  |  |  |  |  |
| 2011 | -0.12^*^ | | [-0.254,0.013] | -0.056 | [-0.526,0.414] | -0.045 | [-0.231,0.14] |
| 2012 | -0.289^***^ | | [-0.451,-0.126] | 0.283 | [-0.332,0.899] | -0.077 | [-0.306,0.153] |
| 2013 | -0.181^*^ | | [-0.388,0.027] | 0.29 | [-0.484,1.064] | -0.157 | [-0.445,0.131] |
| 2014 | -0.287^**^ | | [-0.559,-0.015] | 0.594 | [-0.461,1.65] | -0.164 | [-0.546,0.218] |
| 2015 | -0.173 | | [-0.408,0.062] | 0.674 | [-0.235,1.583] | 0.008 | [-0.321,0.336] |
| 2016 | -0.235 | | [-0.567,0.097] | 1.348^**^ | [0.057,2.638] | -0.157 | [-0.624,0.31] |
| Income |  | |  |  |  |  |  |

^***^ p<0.01, ^**^ p<0.05, ^*^ p<0.1; UEBMI Urban Employees’ Basic Medical insurance; URBMI Urban Residents’ Basic Medical Insurance; All models were adjusted for gender, age group, hospital level, comorbidity, year, and cities’ per capita disposable income.
